# Phylogenetic support values are not necessarily informative: the case of the Serialia hypothesis (a mollusk phylogeny)

**DOI:** 10.1186/1742-9994-6-12

**Published:** 2009-06-26

**Authors:** J Wolfgang Wägele, Harald Letsch, Annette Klussmann-Kolb, Christoph Mayer, Bernhard Misof, Heike Wägele

**Affiliations:** 1 Zoologisches Forschungsmuseum Alexander Koenig, Adenauerallee 160, 53313 Bonn, Germany; 2 J. W. Goethe University, Institute for Ecology, Evolution and Diversity, Siesmayerstrasse 70, D – 60054 Frankfurt am Main, Germany; 3 Ruhr-University Bochum, Faculty of Biology, Universitätsstr., 44370 Bochum, Germany

## Abstract

**Background:**

Molecular phylogenies are being published increasingly and many biologists rely on the most recent topologies. However, different phylogenetic trees often contain conflicting results and contradict significant background data. Not knowing how reliable traditional knowledge is, a crucial question concerns the quality of newly produced molecular data. The information content of DNA alignments is rarely discussed, as quality statements are mostly restricted to the statistical support of clades. Here we present a case study of a recently published mollusk phylogeny that contains surprising groupings, based on five genes and 108 species, and we apply new or rarely used tools for the analysis of the information content of alignments and for the filtering of noise (masking of random-like alignment regions, split decomposition, phylogenetic networks, quartet mapping).

**Results:**

The data are very fragmentary and contain contaminations. We show that that signal-like patterns in the data set are conflicting and partly not distinct and that the reported strong support for a "rather surprising result" (monoplacophorans and chitons form a monophylum Serialia) does not exist at the level of primary homologies. Split-decomposition, quartet mapping and neighbornet analyses reveal conflicting nucleotide patterns and lack of distinct phylogenetic signal for the deeper phylogeny of mollusks.

**Conclusion:**

Even though currently a majority of molecular phylogenies are being justified with reference to the 'statistical' support of clades in tree topologies, this confidence seems to be unfounded. Contradictions between phylogenies based on different analyses are already a strong indication of unnoticed pitfalls. The use of tree-independent tools for exploratory analyses of data quality is highly recommended. Concerning the new mollusk phylogeny more convincing evidence is needed.

## Background

The fact that a clade has a high support in phylogenetic trees does not necessarily imply that (a) the clade is a real monophylum and (b) that the support for the clade is really phylogenetic signal. There are many causes of error. In theory, bootstrap values give no indication of whether there is a systematic problem within the data set [1]. Bayesian support values may be too optimistic, and a bias may cause convergence to an incorrect tree. A "bootstrap support of 100% is not enough, the tree must also be correct" [2]. Furthermore, tree topologies and support values do not show the large differences in information content of data sets, so that practically randomized data may be represented by a well resolved and well supported binary tree [3].

The surprising result obtained by Giribet et al. [4] concerning mollusk phylogeny is essentially a topology for which the information content of the underlying data remained unknown. This is not the only recent publication with this problem (see other examples in [3]), but it is a prominent and interesting case. The analysis was based on sequences from five genes and 108 species, including seven outgroup taxa, suggesting sufficient information was available. Here we show that this is not necessarily the case. A general problem is that despite being available and informative, topology-independent tools for estimation of data quality (e.g. quartet mapping, split decomposition, phylogenetic networks) are not much used.

The tree published by Giribet et al. [4] constitutes a strict consensus topology based on the parsimony criterion and implied alignments obtained by direct optimization [5]. This topology, if accepted as a phylogenetic hypothesis, implies relationships that are highly implausible considering mollusk anatomy and biology. The main dubious inference is the polyphyly of Bivalvia and Gastropoda. Bivalves are highly specialized mollusks with a large number of unique characters such as the laterally compressed body, the bivalved shell with a hinge and ligaments, the loss of the buccal apparatus with radula, acquisition of two special adductor muscles for the shells, special pallial muscles, and a spade-like foot adapted for burrowing [6-10]. Similarly, gastropods are found in two different clades in the published topology, and as in the case of Bivalvia, there are many reasons why this polyphyly is highly improbable [e.g., [6,11-13]]. Another highly improbable grouping is the sister taxon relationship of Caudovofeata and Cephalopoda. This combination has never been suggested before and no apomorphies which would support this monophylum are known to us.

Most of the idiosyncrasies of the published topology are not discussed by Giribet et al. [4]. They focus mainly upon only the "Serialia" clade, composed of Polyplacophora and the monoplacophoran species *Laevipilina antarctica*, and present a new hypothesis for the origin of Monoplacophora. The quality of the data was never discussed, although the single "monoplacophoran DNA was highly degraded" [4] and only an incomplete sequence of *L. antarctica *was obtained (1280 bp of the 28S rRNA). One wonders why only one of the clades in that tree was considered worthy of discussion whereas most parts of the topology are highly implausible, and whether the implied alignments used by Giribet et al. [4] are informative enough to support this hypothesis.

Using methods independent of tree reconstruction, we show that the signal-like patterns in this "largest data set of mollusks ever assembled" are weak, that application of alternative tree-reconstruction methods partly results in alternative hypotheses, and that morphological evidence contradicts the Serialia hypothesis.

## Methods

### Alignments

#### Complete alignment

The multigene data set provided by Dr. Giribet (based on an implied alignment obtained after a POY analysis [5,14] is a multifragment alignment of 108 taxa with many missing data. From the total length of the alignment (9378 bp) more than 30% of the base positions are unsequenced. Additionally, 29% of the base positions are represented by alignment gaps resulting in an alignment with 60% missing data or gaps. For re-analysis of the complete data set, we did not alter the original alignment in the first step. As a second step, this alignment was purified from ambiguous sites after identification of ambiguous and random-like regions with the help of ALISCORE [15], and an additional data set was obtained after elimination of positions with gaps or missing data.

#### 28S rRNA fragment

Since the only information relevant for the placement of the monoplacophoran species is contained within the analyzed 28S rRNA fragment (positions 2959–4254 of the original alignment), we extracted from the original alignment those sequence fragments covering this region. Eight of the 108 species had to be excluded, since no sequence fragments of that area were available for them (see table 1 in [4]). The alignment is 1280 bp long, consisting of 56% missing data (33% non sequenced regions, 23% alignment gaps). Our original intention to use novel software in order to fold the RNAs, align them according to the calculated secondary structure, and apply RNA-models for helical regions was not feasible due to the incompleteness of the available 28S fragments. Sequences were realigned with the Mafft v6.240 program [16,17], which offers various multiple-alignment strategies. For our analysis, we employed the E-INS-i method and default settings for gap opening and gap extension. The length of the resulting alignment was 986 bp. Similar to the procedure for the complete alignment, the 28Sr RNA fragment was also subjected to an analysis with ALISCORE (see below).

### Identification of ambiguous sites

Identification of ambiguous and noisy alignment positions of the complete alignment and of the 28S partition was achieved with ALISCORE[15]. Noisy positions contain nucleotide patterns that cannot be distinguished from randomized ones. The software identifies ambiguously aligned and random-like regions in multiple sequence alignments, and has certain advantages in comparison to G-Blocks [[18]; see also [15]]. The Monte Carlo resampling compares the score of the originally aligned sequences in a given window position with scores of randomly drawn sequences of similar character composition. Hence, ALISCORE provides a formal approach to evaluating sequence alignments and to identifying sections of random similarity caused by saturated sequence divergence, lack of data, and/or alignment ambiguity. The following settings of ALISCORE were used for both alignments: window size was six positions, gaps were treated as ambiguous characters, and pair-wise comparisons were guided by a neighbor joining tree, representing the p-distances of the included taxa.

### Signal-like patterns in alignments and conflict

We subjected the original complete alignment, as well as the original alignment of the 28S rRNA partition to a split-decomposition analysis using SplitsTree Vers. 4.6 [19,20]. Split graphs show more clades than those depicted in a binary tree graph and visualize conflicting evidence. Since we were mainly interested in the structure of the raw data, the neighbornet network [21,22] based on uncorrected distances was most relevant. We also compared graphs based on application of different substitution models, (HKY model: [23]; GTR model: [24]), with uncorrected data, and compared results based on masking of problematic character sets (e. g. gap rich regions). The longest branches visualized in neighbornet graphs were excluded in order to study noise effects that are introduced by long branches.

Split-supporting nucleotide patterns with putative synapomorphies [25,26] were visualised with the SAMS program [3], which allows identification of conserved split-supporting positions without reference to a tree and is therefore independent of model assumptions. This software is not used to construct trees but for exploratory analyses of alignments, especially for visualizing the signal-to-noise ratio. Patterns of supporting positions were identified in the 28Sr RNA fragment of the original alignment, which is the only relevant fragment supporting the Serialia hypothesis.

### Quartet mapping

We used the quartet mapping technique [27] as implemented in *quartm2 *[28] to asses relationships of the *Laevipilina *sequence with four predefined groups of sequences: groups B: Bivalvia, G: Gastropoda, P: Polyplacophora, and S: Scaphopoda. *Laevipilina *as the query sequence is compared to all possible combinations of these three groups of sequences in the way that in each case two predefined groups of sequences, for example B + G, are combined into one. Quartets of sequences are randomly drawn, and support for each of the three possible unrooted topologies of these quartets is calculated. For all analyzed quartets, a unit simplex can be drawn to visualize support for the three different topologies among the four groups of sequences [27,28]. This method has the advantage that quartet mapping directly tests support for an interior branch without any reference to phylogenetic structure within predefined groups. This effectively leads to a reduction of noise.

The analyses were conducted three times based on the original alignment: (A) with all characters, (B) without columns containing gaps and (C) with the data after application of ALISCORE masking. In all three analyses it was apparent that *Laevipilina *fits best the Scaphopoda and Polyplacophora sequences, albeit without strong support. Red circles indicate the mean fraction of simplex points and radius the standard deviation. In all three groups, the mean center of simplex points is within the star like tree area, indicating only weak, if any, signal for a single preferred topology. Exclusion of gap-containing columns and masking the alignment with the ALISCORE approach enhanced signal, but not beyond the star tree like area.

### Bayesian analysis

We used the MrBayes 3.1.2 program [29,30] to infer a posterior probability distribution of topologies and branch lengths of the original alignment and the 28S rRNA partial gene of the original alignment. We applied the substitution model and parameters chosen by Giribet et al. [4], and determined burn-in by inspecting time-series plots of the log posterior to identify the stationary phase. For each run, 10,000 trees were discarded as "burn-in" trees separately, equivalent to 1 million generations. Posterior probabilities were calculated using a 50% majority-rule consensus tree from the set of trees generated in all MCMC runs.

### Maximum likelihood analysis

Maximum Likelihood analyses of the original alignment and the 28S rRNA partial gene alignment were conducted with the parallel Pthreads-based version of RAxML 7.0.4 [31]. Nucleotide substitution was displayed by the GTR model with all model parameters estimated from the data and four categories of gamma distributed rates across sites. Using this model, Maximum Likelihood bootstrap percentages were obtained after 1000 replications.

## Results

### Data quality

Some of the sequences currently deposited in GenBank are mislabelled or based on contamination. Of major importance for the present study is the 28S rRNA *Chaetoderma *sp. AY145397 (Caudofoveata) sequence, which was named *Chaetoderma nitidulum *in Giribet et al. [4]. This sequence is identical to that of the vestimentiferan polychaete *Riftia *(e-value 0.0), hence, Caudofoveata or Solenogastres are not represented at all in the 28S rRNA data set. Where *Chaetoderma *appears in our graphs, this is probably a polychaete sequence.

### Ambiguous sites

Alignment columns with a nucleotide pattern that cannot be distinguished from randomized patterns were filtered out with ALISCORE, leaving only the more conserved sites. In the evaluation of the alignment we consequently treated gaps as ambiguous characters. Due to the large number of missing data and the variability of some gene areas in the complete alignment, only few positions survived the procedure. ALISCORE detected 6303 sites as putative, randomly similar (67,21%). For the 28Sr RNA fragment, 187 positions (19,32%) are putative, randomly similar. Using only conserved sites in phylogenetic analyses does not exclude misleading effects caused by parallelisms and symplesiomorphies [3], but some of the background noise is filtered out.

### Nonparametric split-supporting patterns

#### Complete alignment

**Figure 1:**
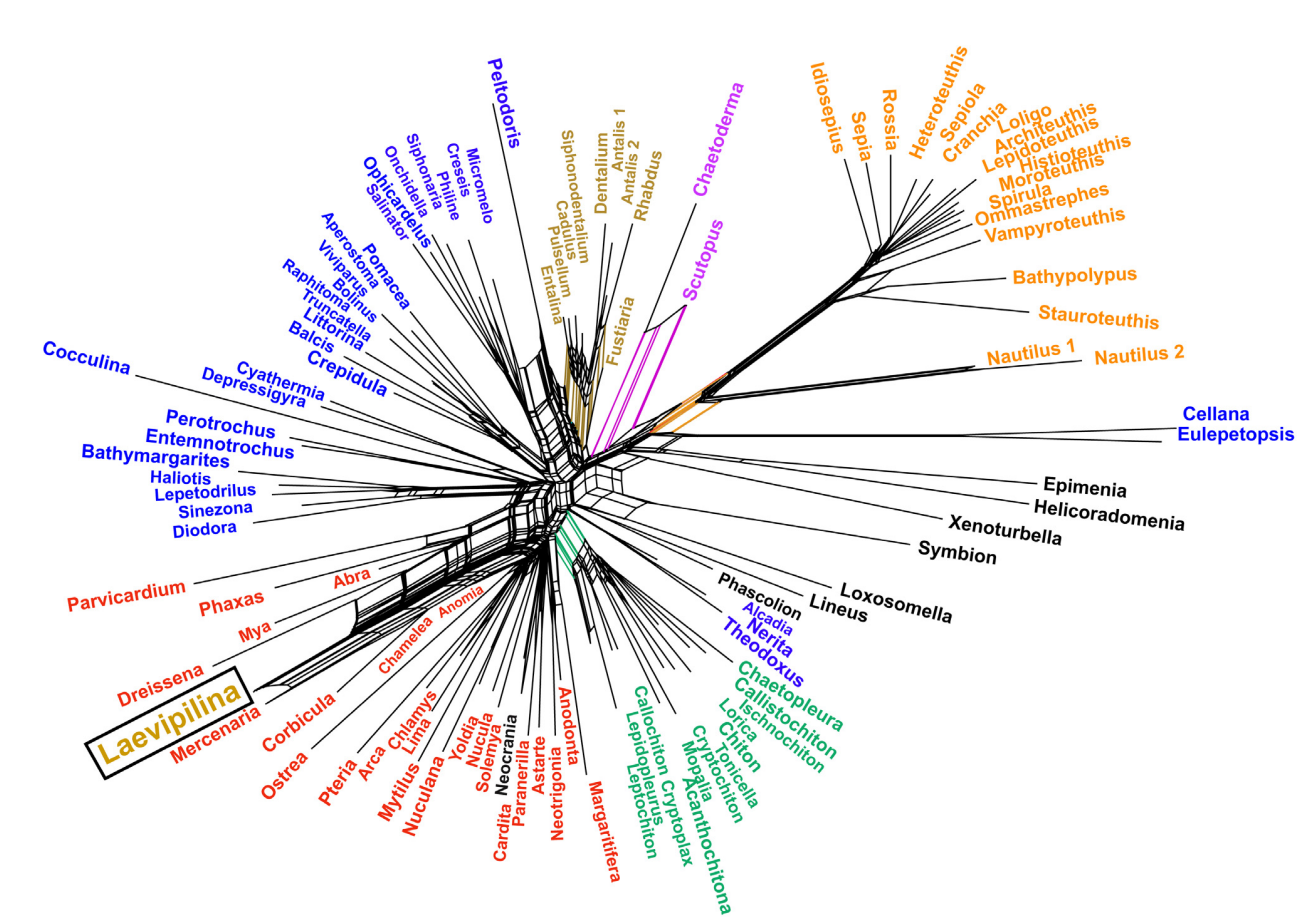
**Neighbornet graph estimated from p-distances with SplitsTree and using the complete alignment from Giribet et al. (2006)**. Color code: Cephalopods are shown in orange, Caudofoveata mauve, Scaphopoda brown, Gastropoda blue, Polyplacophora green. *Laevilipilina *is nested within a subclade of the Bivalvia (red). Note long branches leading to cephalopods and to the gastropods *Cellana *and *Eulepetopsis*, which together form a weak clade probably supported by parallel substitutions. Polyphyly of gastropods and lack of distinct treeness indicates that, in this alignment, there is little conserved phylogenetic signal which is stronger than noise.

For exploratory data analyses we first used the original, complete alignment[4]. Neighbornet graphs constructed from uncorrected distances (Fig. 1: all 9378 positions, 108 taxa, fit value = 93,08) had only few splits supported by distinct edges. The clade Serialia as proposed by Giribet et al. (2006) does not exist in this inference. The monoplacophoran sequence (*Laevipilina antarctica*) is found amidst a cluster of bivalves. The most prominent split separates all cephalopods except the *Nautilus *sequences, which branch off more basally from the cephalopod clade and is also supported as a whole by a set of parallel edges (Fig. 1: taxa and separating edges in orange). The remaining network is dominated by parallelograms, hence it is obvious that the alignment contained many conflicting nucleotide patterns. The signal for monophyly of the Mollusca was not distinct. The Caudofoveata (*Chaetoderma nitidulum *and *Scutopus ventrolineatus *in mauve) are clearly separated from the remaining sequences, and there are short parallel edges for the two clades Scaphopoda and Polyplacophora (Fig. 1, brown and green, respectively). The Gastropoda are scattered over the graph (blue). Two long-branched gastropod sequences (*Cellana *sp., *Eulepetopsis vitrea*) are attracted to the long cephalopod branch. Non-monophyly of Gastropoda and Bivalvia together with a lack of jackknife-support values for the deeper nodes were also attributes of the tree published by Giribet et al. [4]. The lack of support for deeper clades in Figure 1 indicates the absence of a distinct phylogenetic signal for most of the larger species groups.

**Figure 2:**
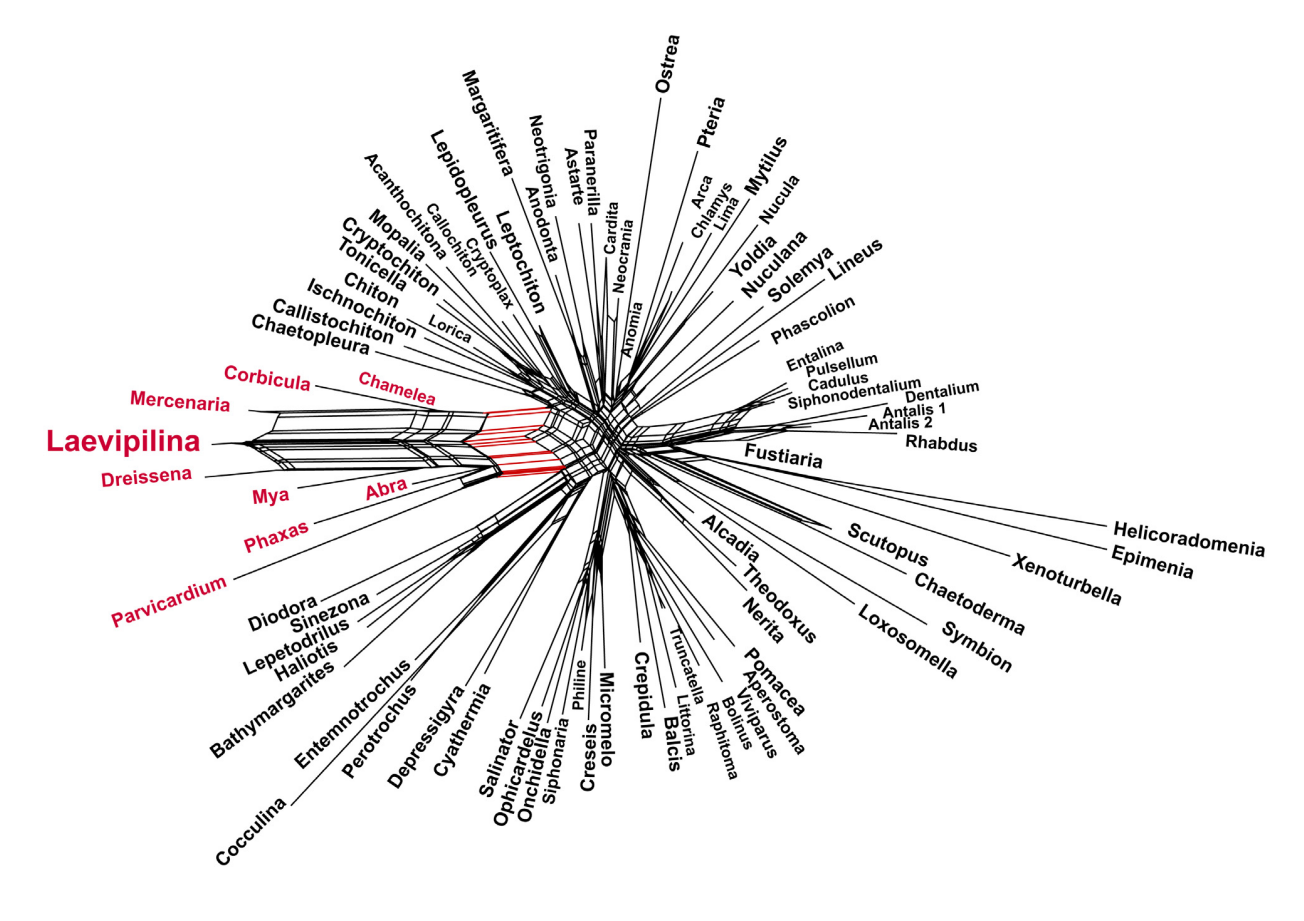
**Neighbornet graph estimated from p-distances with SplitsTree and using the complete alignment from Giribeet et al**. (2006) as in Figure 1, but without long-branch taxa (cephalopods, *Cellana*, *Eulepetopsis*, *Peltodoris*). Neither Bivalvia, nor Gastropoda are monophyletic. The Serialia are not supported.

To reduce the noise in the original data set, we excluded the most conspicuous long branches identified visually in network analyses (cephalopods, and the three gastropods *Cellana *sp., *Eulepetopsis vitrea *and *Peltodoris atromaculata*, see Figs. 1 and Five). This selection (Fig. 2) does not improve the network, treeness is still missing, and there is a set of parallel edges separating a clade composed of the only monoplacophoran species and several Bivalvia species, the latter belonging to the highly derived Euheterodonta clade. Additional exclusion of gaps or application of substitution models altered the length of branches but not the general topology. Obviously, long branches are not the only cause for conflicts in this data set.

#### 28S rRNA fragment

**Figure 3:**
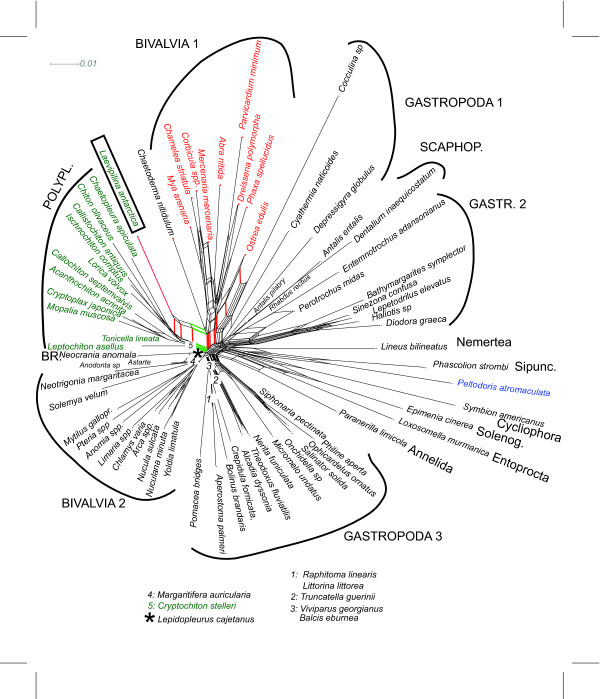
**Neighbornet graph for the 28S portion of the original alignment, the region for which data of *Laevipilina *is available**. A set of short parallel edges supports a split that separates *Laevipilina *and most Polyplacophora (green), but a similar split unites *Laevipilina *with Bivalvia (red). The alignment does not contain a distinct nucleotide pattern supporting the Serialia.

In the network analysis of the original 28S rRNA fragment alignment we do find the Serialia group, at least at first sight, although the polyplacophoran *Lepidopleurus cajetanus *is not part of this clade (star in Fig. 3: placed at base of Brachiopod-Bivalvia 2 split). Exclusion of long-branch taxa (*Cellana *sp., *Eulepetopsis vitrea*, *Creseis *sp. and Cephalopoda) does not alter the network (Fig. 3), but the conflict in the data becomes more obvious. It is important to note that in this analysis, the support for a Serialia clade (excluding *Lepidopleurus*) is comparable to that of {*Laevipilina *and a subgroup of bivalves}, indicated by the length of the edges (green vs. red in Fig. 3). The weight (corresponding to branch length: [21]) for the first split is 0.0049, for the second split 0.0056.

**Figure 4:**
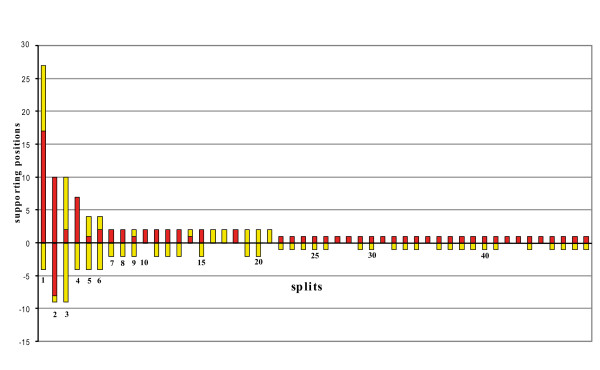
**Spectrum of split-supporting positions as estimated with SAMS**. Column height represents the number of clade-supporting positions, i.e. putative primary homologies. Column parts above the y axis represent the best supported of the two groups of a split, the part below the axis corresponds to the second group. Red: asymmetrical positions (conserved character state only in functional ingroup); yellow: noisy positions (more than one character state in functional in- and outgroup; see Wägele and Rödding 1998a, b). The first ten columns represent the best supported splits and contain the following groupings: 1: *Cellana sp.+ Eulepetopsis vitrea*; 2: Coleoida; 3 Coleoida + *Creseis sp*; 4: *Nautilus pompilius *+ *Nautilus scrobiculatus*; 5: Cephalopoda; 6: Cephalopoda + *Creseis sp*.; 7: *Paranerilla limicola *+ *Chaetoderma nitidulum*; 8: *Chamelea striatula *+ *Corbicula spp *+ *Mercenaria mercenaria*; 9: *Cellana sp.+ Eulepetopsis vitrea *+ Coleoida; 10: *Cellana sp*. + *Eulepetopsis vitrea *+ *Creseis sp*. + *Laevipilina antarctica*. No split was detected that unites *Laevipilina *and Polyplacophora.

**Figure 5:**
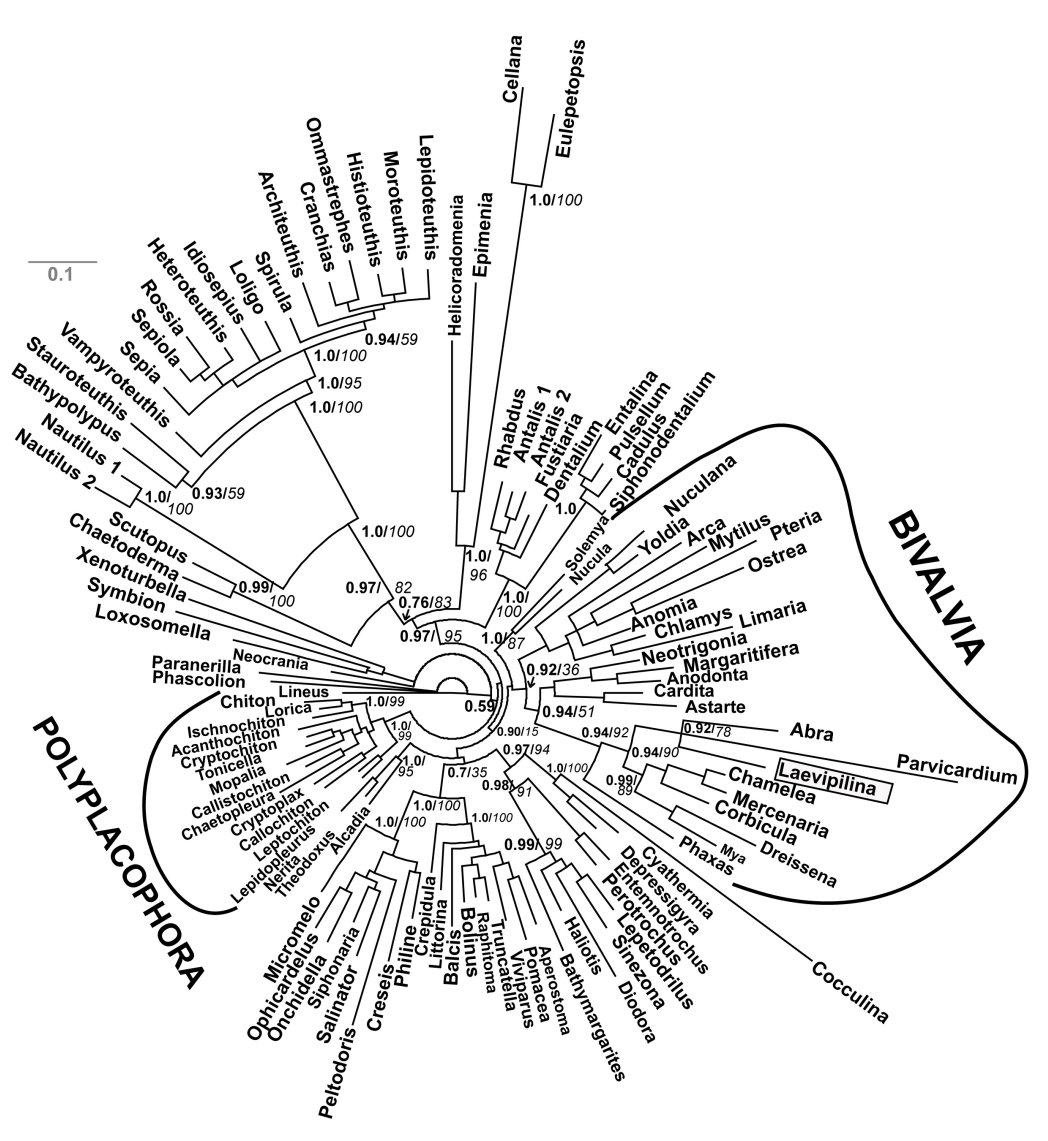
**Bayesian analysis of original alignment (Giribet et al. 2006) after masking of random-like alignment regions with ALISCORE. **.Monophyly of Bivalvia is supported except for the inclusion of *Laevipilina *in this clade (compare also with Figs. 1, 2, 3)

**Figure 6:**
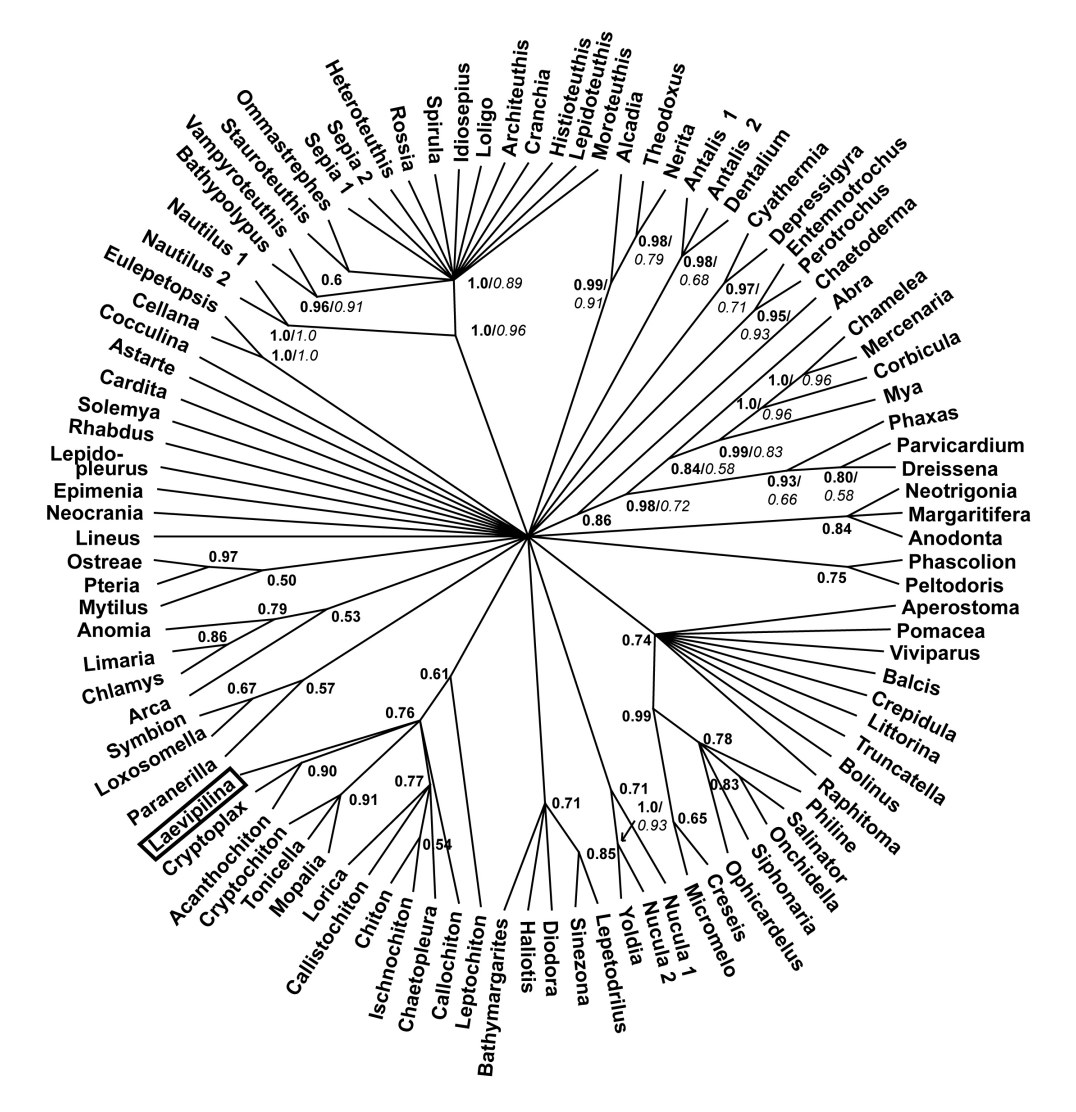
**Bayesian analysis of the realigned 28Sr RNA partition purified with ALISCORE**. Support for Serialia exists, but is negligible.

Application of SAMS was performed in order to identify conserved clade-supporting positions (= putative homologies) for Serialia within the 28S fragment of the original alignment. Note that SAMS does not need a tree. This application represents all splits in an alignment and identifies putative primary homologies. Fig. 4 shows the first 50 splits with the highest support. There are only few splits with distinct underlying nucleotide patterns. The best split contains a clade composed of the two patellogastropods *Cellana *sp. and *Eulepetopsis vitrea *(17 asymmetrical positions and 14 noisy positions), which is also the longest branch in Fig. 5. The next column represents the split between the cephalopod group Coleoida vs. all other taxa, with 10 asymmetrical positions supporting the functional outgroup and 8 conserved positions supporting the functional ingroup (Coleoida). For the more basal nodes of the mollusk tree no conserved nucleotide patterns can be detected (see also Fig. 6). No split with conserved homologies supporting the group {Polyplacophora + *Laevipilina antarctica*} is present.

### Quartet mapping for the 28S rRNA fragment

**Figure 7:**
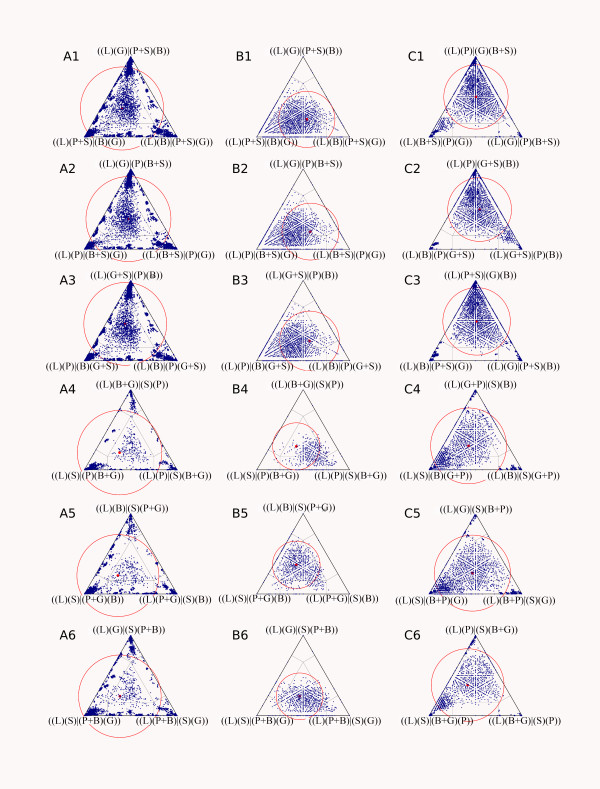
**Visualizing phylogenetic structure of alignments via quartet mapping (Nieselt-Struwe and von Haeseler, 2001)**. Dots in a corner of a triangle represent high support for only one of the three topologies that can be constructed for a quartet of taxa. Dots in the centre represent a star-like topology, and the rest of the triangle stands for intermediate situations. Red circles indicate placement of the mean fraction of points. In all cases the majority of quartets are near the star-tree region, indicating little or no phylogenetic signal. The studied combinations are: A1–6: Original alignment of Giribet et al. (2006) with all characters. B1–6: Same alignment after exclusion of columns with gaps or missing data. C1–6: Same alignment after masking with the ALISCORE approach. For each alignment, the association of *Laevipilina *with all six possible variants of pairs of higher mollusc taxa were examined (see text). B = Bivalvia, G = Gastropoda, L = *Laevipilina*, P = Polyplacophora, S = Scaphopoda.

The analyses of the 28S region of the original alignment were executed three times: (A) with all characters, (B) without gap-containing columns, and (C) with the data after application of ALISCORE masking. Accumulation of dots in triangle corners and absence of dots in the central region of triangles are indications for phylogenetic structure of the data set. In all three analyses (Fig. 7) it is apparent that *Laevipilina *fits best the Scaphopoda and Polyplacophora sequences (triangle corners with groups {(L)(S)} and {(L)(P)}), albeit without strong support. Red circles indicate the mean fraction of simplex points and the radius represents the standard deviation. In all three groups the mean center of simplex points (red dot) is within the star like tree area, indicating only weak if any signal for a single preferred topology. Excluding of gap-containing columns and masking the alignment with the ALISCORE approach enhanced signal, but not beyond the star tree area.

Note that exclusion of positions with gaps or missing data eliminates most of the informative sequence positions – a consequence of the large unsequenced regions in many sequences of this data set. Exclusion of random-like positions with ALISCORE retains more information.

### Tree reconstruction

#### Complete alignment

Reanalysing the original alignment under maximum likelihood and Bayesian assumptions, gave similar results as those of Giribet et al [4], with similar high support values (not shown). However, because many regions of the alignment contained a high proportion of missing data and many regions could not be aligned unambiguously, we repeated the phylogenetic analyses with a purified and necessarily smaller data set by excluding all positions identified by ALISCORE as random-like. As seen in Fig. 7, ALISCORE conserves more structure in the data (quartet series C) than a flat deletion of all sequence positions containing gaps (quartet series B).

This resulted in different topologies in both the Bayesian and the maximum likelihood analyses (Fig. 5); in contrast to previously published trees [4,32,33], but in agreement with the neighbornet analysis, all bivalve taxa clustered together, with only *Laevipilina antarctica *nested within that clade.

#### 28S rRNA fragment

Phylogenetic analyses of the 28S rRNA fragment of the original alignment exhibited incongruent results: Serialia has been recovered by applying Bayesian inference (Fig. 6) but with a low posterior probability (0.61) and the polyplacophoran *Lepidopleurus cajetanus *was not a part of the Seralia clade (in agreement with Neighbornet analyses). In the Maximum Likelihood analysis, the gastropods *Cellana *sp. and *Eulepetopsis vitrea *nested within the Serialia clade (no bootstrap support), but again, *Lepidopleurus cajetanus *was not included in this group.

After implementation of ALISCORE and exclusion of ambiguous sites, additional analyses (Maximum Likelihood and Bayesian inference) were performed with the shorter fragment. Serialia in the sense of Giribet et al. [4] were recovered applying the Bayesian inference, albeit with a posterior probability of only 0.75 and without bootstrap support. In the Maximum Likelihood analysis, the gastropod *Peltodoris atromaculata *was nested within the Serialia.

It is noteworthy that all analyses of the 28S rRNA fragment, irrespective of exclusion or inclusion of ambiguous sites, resulted in highly unresolved consensus trees, indicating lack of phylogenetic signal in this particular fragment (see Fig. 6).

## Discussion

### Data quality and phylogeny inference

The notable incongruence among trees in the published literature clearly indicates that good resolution of and support for clades in published topologies are no proof of the reliability of the data and methods of analysis. For example, crustaceans are polyphyletic in [34] but paraphyletic in [35]. Tardigrades are the sister taxon of Nematoda in [36], but the sister group of Onychophora and Euarthropoda in [37]. Since, in each case, at least one of these pairwise incompatible inferences must be incorrect, it is legitimate to ask which data have a better signal-to-noise ratio. It has also been demonstrated in simulations that false clades with high support may be found in inferred trees that do not correspond to the original topology [e.g. [38,39]]. Therefore, the fact that the Serialia clade of Giribet et al. [4] had a jackknife support of 95 in the parsimony analysis is not necessarily evidence for the discovery of a new and distinct phylogenetic signal.

In the case of the mollusk phylogeny discussed here, the original alignment seems to be rich in data at first glance (five genes and 108 species were used), however, the quality of these data is very difficult to estimate if one only reads the publication. Since Giribet et al. obtained only a partial sequence (about 1.2 kb of 28SrRNA) of the monoplacophoran *Laevipilina antarctica*, and the placement of the monoplacophoran can only be the result of similarities shared in the 28S partition of the data set, the relevant information is limited.

One may argue that gaps and missing data do not bias phylogenies, but this depends on the patterns of missing characters. Hartmann and Vision [40] showed by simulation studies with incomplete alignments that parsimony algorithms in particular, as applied in POY, had the lowest accuracy in finding the correct tree and the highest sensitivity for patterns of missing data. Topological disagreement with the original tree of the simulation (range from 0 showing complete agreement to 1 showing complete disagreement) approached the median value of 0.4 in alignments with 60% missing data (similar to the alignments in [4]), but 1.0 only in correctly aligned simulated data (very unlikely in the alignments in question).

Giribet [41] argued that a primary homology (positional homology in the starting alignment) is irrelevant if it does not comply with the congruence test (fit of characters to a tree), hence Giribet et al. [4] rely on phylogenies inferred with POY. Using POY means that criteria for primary homology are not separated from optimality criteria used for tree inference (co-optimizing topology and homology), or, in other words, that homology hypotheses are adapted with the method to optimize results, which leads to circular reasoning. Quality criteria based on empirical and topology-independent evidence, such as variability and similarity of sequence regions and fit of positions to a secondary structure model, are not considered. This is a fundamental difference from our approach: In any empirical science, data quality can and should be evaluated prior to analyses that aim at hypotheses testing. It should be stressed that evaluation of data quality and the use of alignments to infer an hypothesis can be usefully treated as two *independent *steps [e.g. [20,39,42]], although a discussion of this point is beyond the scope of the present study. For the relevance of primary homologies see [43-50]. For problems with POY see [51-53].

The key question raised by Giribet et al.[4] – the position of Monoplacophora within Mollusca,- was based only on a fragment of the 28S rRNA gene (1280 bp). Moreover, a rather high proportion of taxa is represented by short sequences, 18% of which are represented by less than 350 bp. This raises the question of whether the available information is really sufficient to support radically new ideas such as the Serialia hypothesis. To answer this we used different tools for an *a priori *data exploration (= prior to traditional tree inference).

#### A priori analyses

Phylogenetic networks are derived from the split decomposition method originally described by Bandelt and Dress [54]. Networks show support for groups of sequences even when they are mutually incompatible and visualize edge-lengths for signal-like patterns and contradictions [3,21,22,55-59]. If networks are tree-like, one can assume that phylogenetic signal dominates in the data set [42]. Figs. 1, 2 and 3 show long terminal branches connected by networks with short edges, thus indicating conflict and lack of distinct split-supporting nucleotide patterns for deeper nodes of the phylogeny.

In Fig. 1, some clades with distinct elongate stems are present (certain gastropod groups, cephalopods, Caudofoveata, Solenogastres), but these are not relevant to the discussion of the deeper mollusk phylogeny. Interestingly, the monoplacophoran sequence shares character states with bivalves (Figs. 1, 2, 5) and Polyplacophora (Figs. 3, 6).

Few of the groupings are separated by distinct splits, i.e. by sets of parallel edges that are longer than those of conflicting splits. This observation is congruent with the spectrum of split-supporting patterns (Fig. 4), which is obtained with a different method but nevertheless shows the same signal-to-noise relation: cephalopods conserve shared character states, but the Serialia do not appear among the 50 best supported splits. The third method we used, quartet mapping, is an entirely different tool but the results it gave are similar (Fig. 7): there is no phylogenetic structure that allows an unequivocal placement of *Laevipilina*.

Although all of these tools can be improved to refine their ability to identify signal-like patterns, it is evident that their use shows congruent results. The large number of conflicting patterns, which are not visible when analyses are restricted to conventional tree inference, call for caution in propagation of new hypotheses.

#### The Serialia in tree topologies

Our tree-reconstruction analyses of the original dataset supported a Serialia clade only with the complete alignment, as was done in the original publication [4]. However, *a priori *network analyses as well as tree reconstruction with the purified alignment (after masking problematic regions with ALISCORE, Fig. 5) contradicted this hypothesis because *Laevipilina antarctica *is nested within the bivalves. Moreover, monophyly of Serialia is only achieved when analysing the 28S rRNA partition after exclusion of ambiguous sites and with very low Bayesian support (Fig. 6), which is unacceptable as clear phylogenetic signal. One also has to keep in mind that Bayesian support may be too optimistic [60]

Biologists often assume that it is sufficient to use correct substitution models for phylogenetic reconstruction. However, missing signals cannot be compensated for even with the best model. Missing signals due to signal erosion have been documented in older radiations of the metazoan tree [61-63] and data transformation using models may even increase the level of noise when deep phylogenies are studied [42].

#### Morphological characters

A cladistic analysis of morphological characters is beyond the scope of the present study, but documented homologies can contradict hypotheses even without inferring a tree. For example, the fine structure of feathers (as evidence for homology of the plumage) would obviously not be compatible with polyphyly of feathered organisms (birds). We therefore briefly review morphological characters as additional sources of information. For discussion of homology of morphological characters we refer to the malacological literature (see below).

Giribet et al. [4] stated that "the disparity of mollusk body plans is so great that it is quite difficult to find a single trait shared by all seven classes of mollusks" and that "preconceived ideas on mollusk relationships ...rely almost entirely on shell morphology". This argument implies that morphology is useless for establishing the monophyly of mollusks, yet many unique traits are shared by all larger mollusk taxa in their basic pattern. Some of these attributes were mentioned in the introduction and additional examples are discussed below. The monophyly of Mollusca is supported by homology of mantle and a mantle cavity containing at least one pair of gills, the ventral "foot", the presence of a dorsal heart with paired auricles, a bilateral nervous system with two pairs of conspicuous longitudinal nerve cords with major commissures only anteriorly, and the presence of rhogocytes. The radula is a unique mollusk character and its secondary absence in particle- or filter-feeding species is easily explained [6] (see latest review of Haszprunar et al. [64]).

The idea that Monoplacophora might be derived from Polyplacophora conflicts with the Conchifera hypothesis, which places extant Monoplacophora (Neopilinida) at the base of the Conchifera, whereas Polyplacophora are usually regarded as the sister group of Conchifera [6,64-68]. In the few morphologically based phylogenetic analyses available to date, this position is confirmed or at least not contradicted [6,68].

Giribet et al. [4] contend that serially repeated gills and the eight sets of dorsoventral retractor muscles are a synapomorphy of Monoplacophora + Polyplacophora. They do not discuss the characters shared by Conchifera, although these cannot be ignored: Conchifera (comprising Monoplacophora, Gastropoda, Cephalopoda, Bivalvia and Scaphopoda) have a massive shell that protects the dorsal visceral mass and covers a larger dorsal area than the serial shells of Polyplacophora. In addition, the outer mantle surface protected only by cuticle and single calcareous spiculae in non-conchiferans is covered by the single massive shell in conchiferans. The suprarectal commissure of the nervous system seen in non-conchiferans is replaced by a subrectal commissure in Conchifera. Conchifera are further characterized by typical statocysts near the pedal ganglion. Haszprunar [64] names another synapomorphy of the Conchifera, namely, the cilia with a single ciliary root, rather than two ciliary rootlets typical of metazoans, including Solenogastres, Caudofoveata and Polyplacophora.

The presence of eight dorsoventral retractor muscles in extant Monoplacophora, regarded by Giribet et al.[4] as a synapomorphy shared with Polyplacophora, can be interpreted as a plesiomorphic homology inherited from the common ancestor of all Conchifera and Polyplacophora (the Testaria or Eumollusca hypothesis [e.g. [65,69,70]]). Some fossil bivalves with eight pairs are known [71,72] and less derived bivalves still show six pedal retractor muscles. The Polyplacophora retain a mobile dorsal exoskeleton composed of eight small shells. According to this view, after fusion of these single shells in the stemline of Conchifera, the eight pairs of rectractor muscles were retained in the most primitive Conchifera, of which the Neopilinida survive. Giribet et al. [4] did not mention that the dorsoventral muscles of *Neopilina *already show signs of simplification: Polyplacophora have *two *pairs of muscles arranged in tandem for each dorsal shell, whereas in the well studied *Neopilina *these are fused – a probable consequence of the simpler shell configuration. It can be assumed that this simplification continued during conchiferan evolution and led to muscle reductions in higher evolved Conchifera [6,65,66,73].

This hypothesis resolves the conflict between homologies shared by Monoplacophora and Polyplacophora on one hand, and homologies present only in Conchifera on the other hand. The first set of homologies consists of plesiomorphies, whereas the second consists of apomorphies of Conchifera. Some similarities of the pharyngeal area of Monoplacophora to the pharynx of chitons as described by Wingstrand [74] are probably homologous (glandular epithelium of subradular sac, similarity of salivary glands, radular vesicles), but it is not clear if these are plesiomorphies or apomorphies and which variations occur in other Conchifera. Giribet et al. [4] mentioned traces of seriality that are seen in different mollusk taxa but did not offer an explanation for this observation. In the traditional understanding of mollusk phylogeny these characters are not enigmatic; some are independent of the shell-adductor system (spicules on caudofoveate larvae) and offer no motive to search for segmentation, whereas others (gills and nephridia in primitive cephalopods) can be interpreted as remnants of the seriality inferred for the last common ancestor of Polyplacophora and Conchifera.

Giribet et al [4] pointed out that shell formation of Neopilinida differs from other Conchifera and concluded that this indicates non-homology. Haszprunar and Schäfer [75] indicated that the foliated layer of nacre is not homologous to the nacre of gastropods. However, these authors also referred to Poulicek and Jeuniaux [76], who considered the microstructure and the composition of the chitinous organic matrix of the neopilinid shell to be more similar to other conchiferans than to polyplacophorans.

## Conclusion

All our analyses indicate that the rejection of the traditional views about mollusk phylogeny by Giribet et al [4] was premature and support in their data set for Serialia is not higher than for alternative hypotheses. We encourage a more critical investigation of molecular data prior to tree reconstruction and the use of analytical methods that detect incongruencies. Problems created by missing data on a large scale have to be addressed in much greater detail, which is especially called for in view of the oncoming floods of EST analyses and other genomic data sets. In order to avoid premature conclusions it is also important to discuss evidence available from other sources, in this case – from comparative anatomy of mollusks. Above all, data quality and completeness should be transparent.

## Competing interests

The authors declare that they have no competing interests.

## Authors' contributions

Split decomposition and phylogenetic network analyses were performed by JWW and CM, application of Aliscore, quartet mapping and Bayesian inferences by HL and BM, morphological data were discussed by AKK, HW and JWW. All authors contributed to the discussion of the results and the preparation of the manuscript.
